# L-histidine inhibits production of lysophosphatidic acid by the tumor-associated cytokine, autotaxin

**DOI:** 10.1186/1476-511X-4-5

**Published:** 2005-02-28

**Authors:** Timothy Clair, Eunjin Koh, Malgorzata Ptaszynska, Russell W Bandle, Lance A Liotta, Elliott Schiffmann, Mary L Stracke

**Affiliations:** 1 Laboratory of Pathology, National Cancer Institute, National Institutes of Health, Bethesda, Maryland 20892, USA

## Abstract

**Background:**

Autotaxin (ATX, NPP-2), originally purified as a potent tumor cell motility factor, is now known to be the long-sought plasma lysophospholipase D (LPLD). The integrity of the enzymatic active site, including three crucial histidine moieties, is required for motility stimulation, as well as LPLD and 5'nucleotide phosphodiesterase (PDE) activities. Except for relatively non-specific chelation agents, there are no known inhibitors of the ATX LPLD activity.

**Results:**

We show that millimolar concentrations of L-histidine inhibit ATX-stimulated but not LPA-stimulated motility in two tumor cell lines, as well as inhibiting enzymatic activities. Inhibition is reversed by 20-fold lower concentrations of zinc salt. L-histidine has no significant effect on the Km of LPLD, but reduces the Vmax by greater than 50%, acting as a non-competitive inhibitor. Several histidine analogs also inhibit the LPLD activity of ATX; however, none has greater potency than L-histidine and all decrease cell viability or adhesion.

**Conclusion:**

L-histidine inhibition of LPLD is not a simple stoichiometric chelation of metal ions but is more likely a complex interaction with a variety of moieties, including the metal cation, at or near the active site. The inhibitory effect of L-histidine requires all three major functional groups of histidine: the alpha amino group, the alpha carboxyl group, and the metal-binding imidazole side chain. Because of LPA's involvement in pathological processes, regulation of its formation by ATX may give insight into possible novel therapeutic approaches.

## Background

Lysophosphosphatidic acid (LPA) is both an intracellular and an extracellular signaling molecule that affects biological processes such as cell proliferation, rescue from apoptosis, cell migration, neurite retraction, wound healing, platelet aggregation and vascular remodeling [1]. As a cytokine affecting such varied and important functions, LPA production is normally tightly regulated. Its dysregulation is implicated in a number of pathophysiological states, including certain cancers and atherogenesis. Intracellularly, LPA is produced by calcium-dependent and calcium-independent phospholipase A2, acting on phosphatidic acid. Most of the extracellular LPA appears to be produced by a two-step process: production of lysophosholipid from phospholipids by the action of phospholipase A1 or A2, followed by conversion to LPA by the plasma enzyme lysophospholipase D (LPLD) [2,3]. Recently, this plasma LPLD has been shown to be identical to autotaxin (ATX, NPP2) [4,5].

ATX was originally purified as a potent tumor cell motogen [6], an effect that appears to be mediated by LPA [7] acting through the LPA1 receptor [8]. Recent studies have revealed that ATX/LPLD not only hydrolyzes lyso-phosphoglycerolipids to form LPA, but also hydrolyzes sphingosylphoshorylcholine (SPC) to produce sphingosine-1-phosphate (S1P) [9]. S1P can stimulate or inhibit cellular migration, depending upon the context of receptor expression [10]. Therefore, ATX can produce either agonists or antagonists of cell migration. In addition, ATX has been shown to stimulate tumor aggressiveness and to be over-expressed in certain malignancies [11-13]. The link between ATX and its putative 'mediator' LPA has led us to investigate possible mechanisms of regulating the enzymatic action of ATX in generating LPA.

ATX is a member of the nucleotide pyrophosphatase and phosphodiesterase (NPP) family of enzymes. The NPPs are part of the superfamily of alkaline phosphatases, metalloenzymes in which the active site is characterized by histidine residues coordinated around central divalent cations and by a serine, threonine, or cysteine residue, which is utilized to form an intermediate during the reaction [14,15]. Site-directed mutagenesis of human ATX established that a specific residue, T210, [7,16] and 3 histidine residues [7,9], corresponding to similar loci in other members of the alkaline phosphatase superfamily, were essential for the motility and enzymatic activities of ATX.

Histidine has been implicated as a requirement for many metalloenzymatic reactions, presumably by virtue of its imidazole moiety. Reversible reagents such as diethylpyrocarbonate, which react with imidazole, inhibit these enzymes [17]. Such findings led us to investigate whether histidine itself and some of its derivatives could inhibit the activities of ATX, perhaps by destabilizing the putative metallic cation-imidazole complex at the active site of ATX. Since the migratory effects of ATX depend upon its ability to generate LPA or S1P, focusing upon ATX as a target for regulation of tumor cell motility presents an attractive strategy for therapeutic intervention in metastasis.

## Results

### Effect of L-Histidine upon Cell Motility

ATX, isolated as a motility-stimulating protein, is a member of the NPP family of metalloenzymes. Both its motogenic and its enzymatic activities appear to require the same active site since both are abolished when T210 [16] or any of three histidines (H316, H360 or H476) are altered by site-directed mutagenesis [7]. Because of the crucial role that these histidines play in the ATX activities, we tested the effect of adding exogenous L-histidine upon ATX-stimulated cell motility.

**Figure 1:**
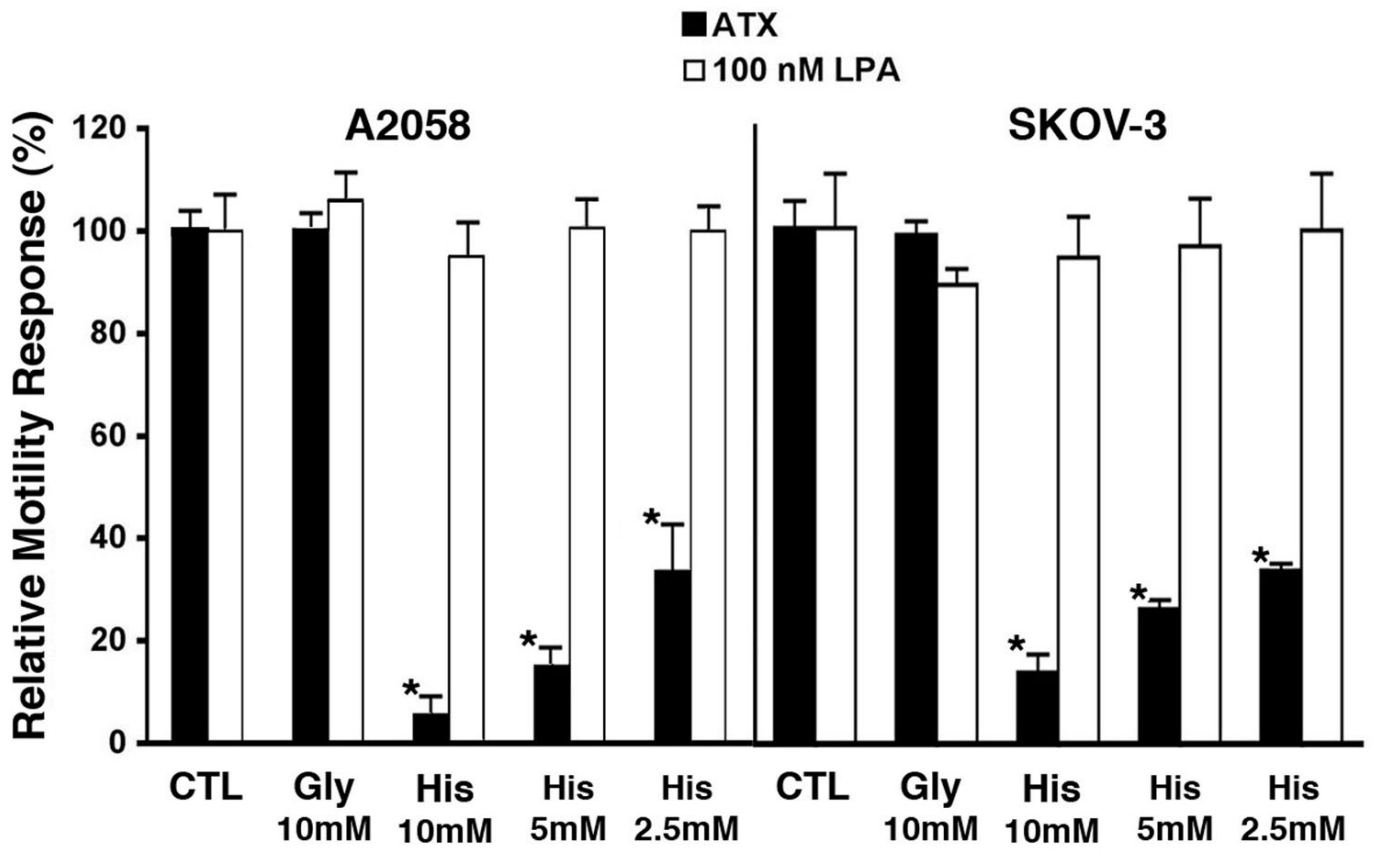
**Effect of histidine on ATX-stimulated motility**. The modified Boyden chamber motility assays are described in Materials and Methods. L-Histidine inhibits the motility response of A2058 and SKOV-3 cells to ATX but not to LPA (concentrations shown). All data are shown as Mean ± SEM. Means were analyzed utilizing the ANOVA/Tukey's post-test (GraphPad Prism, San Diego, CA): * (p < 0.001) for histidine-treated vs. untreated controls.

Fig. 1 shows that L-histidine inhibited ATX-stimulated migration of both A2058 human melanoma cells and SKOV-3 human ovarian carcinoma cells in a concentration-dependent manner. Addition of 10 mM L-histidine to motility assays resulted in a 90 – 95% reduction in stimulated motility, whereas equivalent concentrations of control amino acids, glycine or alanine (data not shown), had no effect. In contrast, adding the same concentration of L-histidine failed to inhibit LPA-stimulated motility. These concentrations of L-histidine did not affect cell viability in either cell line, as measured by Trypan Blue exclusion. Whether the inhibitor was added to both wells of the assay (i.e. to cells and to chemoattractant) or just to the lower well (chemoattractant) did not affect the result.

**Figure 2:**
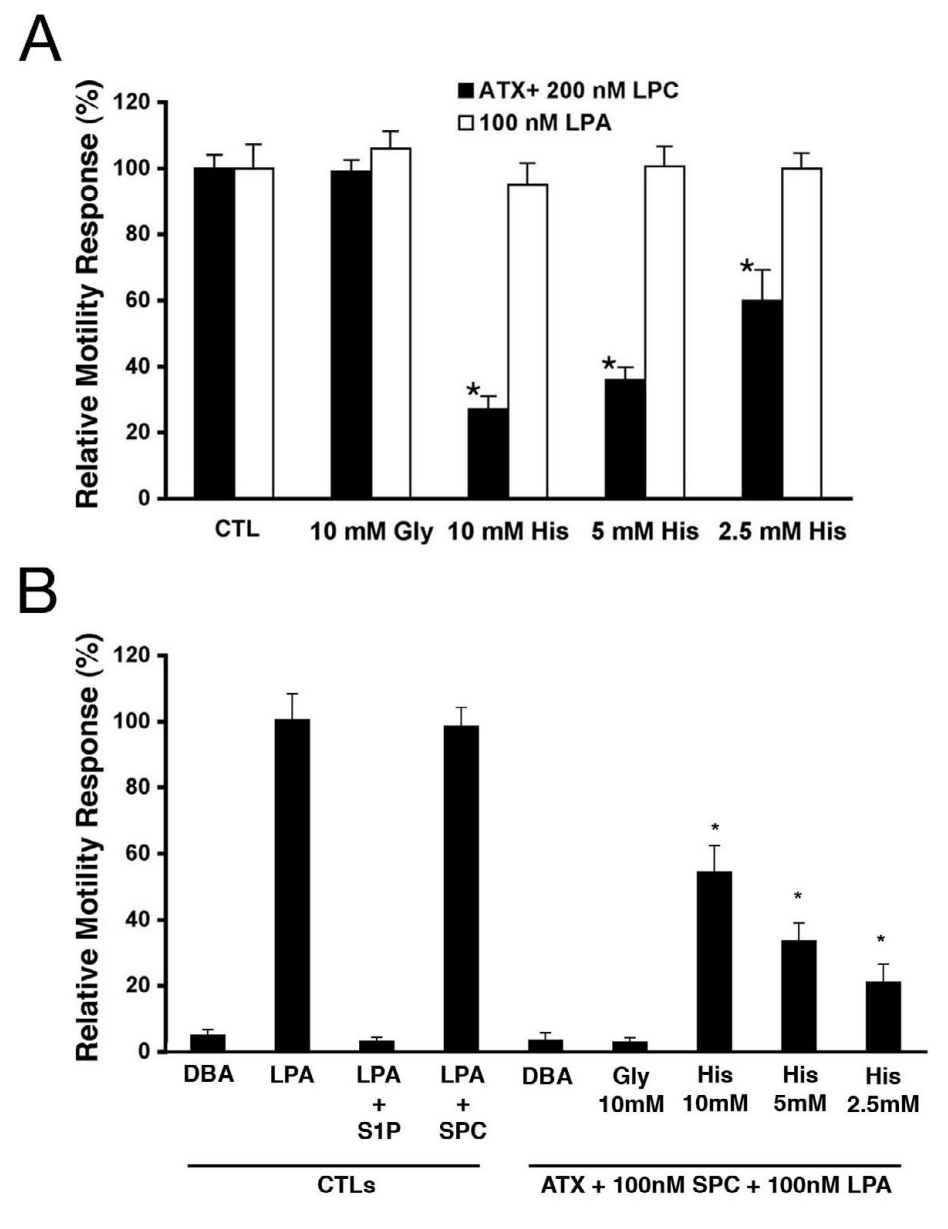
**Histidine appears to act by inhibiting the ATX LPLD activity**. The modified Boyden chamber motility assays with A2058 cells are described in Materials and Methods. In these assays, pre-incubations were in DBA media for 3 hr at 37°C. A) **L-histidine decreases formation of a chemoattractant by ATX**. Shown concentrations of L-histidine were pre-incubated with ATX plus 200 nM LPC, then ATX was heat-inactivated prior to utilizing the resulting mixture as chemoattractant in a motility assay (solid black bars). Data are compared to identical treatments of 100 nM LPA as chemoattractant (white bars). B.) **L-histidine decreases formation of a motility inhibitor by ATX**. Shown concentrations of L-histidine were pre-incubated with (ATX + 100 nM SPC + 100 nM LPA). ATX was heat inactivated prior to utilizing the mixture as chemoattractant. Controls are shown for comparison: DBA, 100 nM LPA, (100 nM LPA + 100 nM S1P), (100 nM LPA + 100 nM SPC). All data are shown as Mean ± SEM. Means were analyzed utilizing the ANOVA/Tukey's post-test (GraphPad Prism, San Diego, CA): * (p < 0.001) for histidine-treated vs. the appropriate untreated control.

ATX was pre-incubated with 200 nM LPC with or without addition of various concentrations of L-histidine, then heated to abolish its enzymatic activity [9] and added to the bottom chamber of motility assays utilizing A2058 cells as responders. As seen in Fig. 2A, addition of L-histidine to the pre-incubation mixture resulted in a 75% inhibition of motility for A2058 cells at concentrations similar to those utilized in Fig. 1. These data, which were identical in SKOV-3 cells (data not shown), suggest that the L-histidine acts upon the ATX-catalyzed LPLD reaction to produce its inhibitory effect by preventing the formation of LPA from LPC. Previously, we have shown that SPC is an alternate substrate for ATX: pre-incubation of SPC with ATX, followed by heat-inactivation of ATX, resulted in production of the inhibitor of LPA-stimulated A2058 cell motility, S1P [9]. As seen in Fig 2B, inclusion of 20 mM L-histidine in the ATX + SPC pre-incubation step prevented the formation of an inhibitor of A2058 cell motility. L-histidine therefore appears to inhibit the hydrolysis of both glycerophospholipids and phosphosphingolipids.

### Effects of Histidine and Metal ions upon ATX Activities

The PDE activity of the NPPs has been shown to be dependent upon the presence of metal ions [15]. Tokumura and co-workers [18] demonstrated a similar requirement for a metal cation in the action of LPLD. Both Co^++ ^and Zn^++ ^were markedly effective in restoring enzymatic activity to LPLD in EDTA-treated plasma. In fact, addition of Co^++ ^to EDTA-treated heparinized plasma resulted in a recovery from 37% of the untreated value in the presence of EDTA alone to 154% in the presence of Co^++ ^and EDTA. Since histidine plays an important structural role in the catalytic process, we determined first that addition of free histidine has an inhibitory effect upon ATX-catalyzed hydrolysis of both nucleotide and lyso-phospholipid substrates. After establishing the concentration requirements for histidine inhibition, we next examined the capacity of different metal cations to reverse this inhibition. Finally, we determined how histidine and metal cations affect the kinetics of the LPLD reaction.

**Figure 3:**
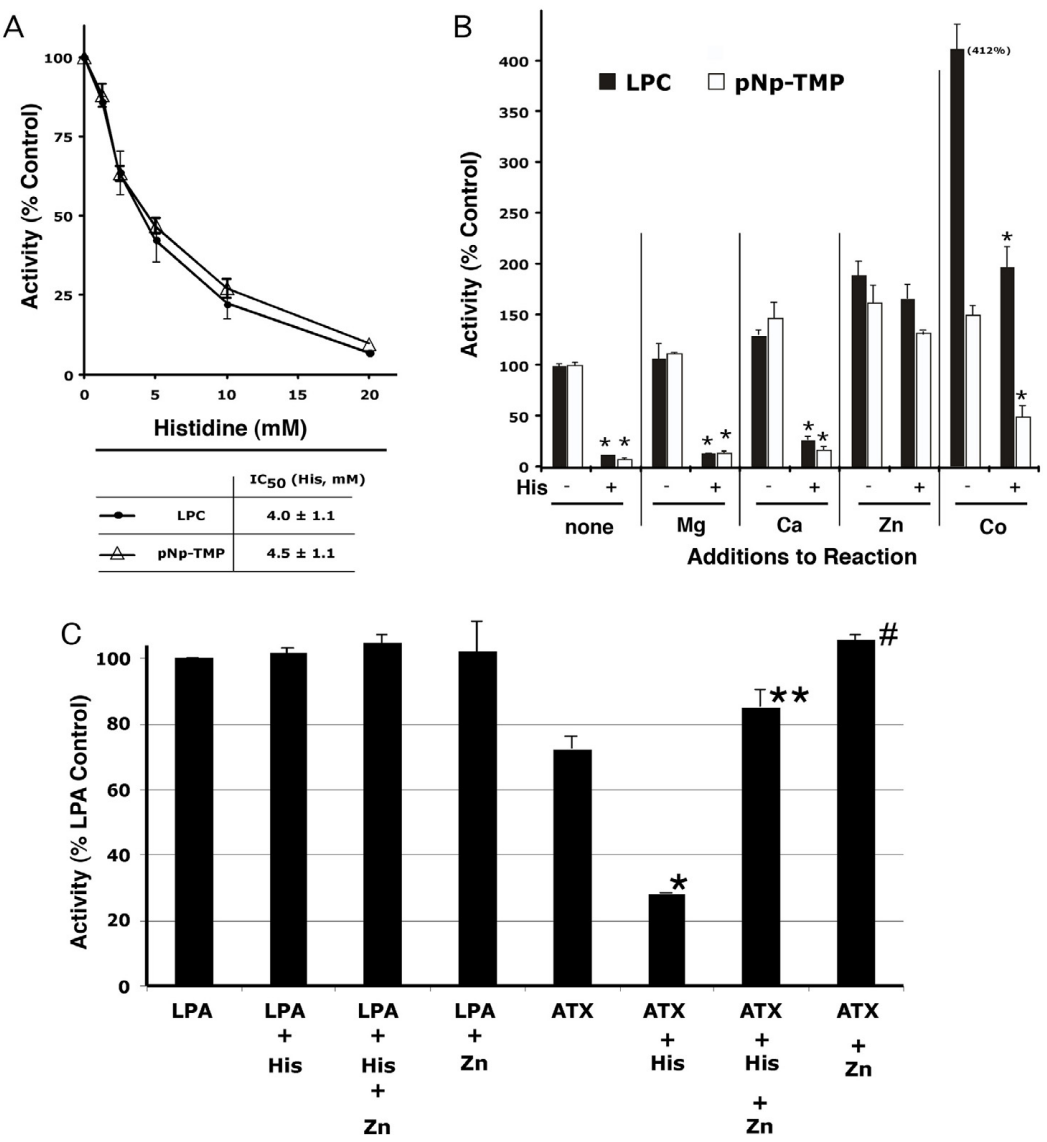
**Histidine inhibition of ATX enzyme activities and sensitivity to zinc**. For these reactions, 1 mM LPC served as substrate for LPLD activity and 1 mM pNp-TMP as substrate for 5'-nucleotide PDE activity; approximately 6 pmoles of ATX was added to each reaction. A.) Product was measured in the presence of variable concentrations of L-histidine. Data are shown as Mean ± SEM. The two reactions were not significantly different from each other. B.) Different metal cations (0.5 mM) were compared for their capacity to abrogate L-histidine (10 mM) inhibition of enzymatic reactions. C.) Effect of Zn^++ ^on LPC- and ATX-stimulated motility. Utilizing A2058 cells as responders and either ATX or 25 nM LPA as chemoattractant, 20 mM histidine or 0.25 mM ZnSO4 or both were added to the bottom wells throughout the motility assay. Data are shown as Mean ± SEM. Means were compared with ANOVA/Tukey's post-test (GraphPad Prism, San Diego, CA). For B), comparisons were between histidine-treated vs. the equivalent untreated reactions with * (p < 0.001). For C), comparisons were as follows: * (p < 0.001) vs. ATX alone, ** (p < 0.001) vs. ATX + His, # (p < 0.01) vs. ATX alone.

As seen in Fig. 3A, histidine inhibited both LPLD (with LPC as substrate) and PDE (with p-Nitrophenyl-TMP or pNp-TMP as substrate) activities of ATX in a dose dependent manner. At a concentration of 20 mM, this inhibition was virtually complete for both reactions. The normalized curves coincided closely with each other and the IC50 values (4.0 ± 1.1 mM and 4.5 ± 1.1 mM for LPLD and PDE activities, respectively) were not significantly different from each other.

We tested the effects of a number of divalent cations in both the presence and absence of histidine upon the PDE and LPLD activities of ATX (Fig. 3B). Neither Mg^++ ^nor Ca^++ ^significantly reversed the inhibitory effect of histidine although Ca^++ ^alone enhanced both reactions by at least 30%. However, Zn^++ ^and Co^++ ^each significantly overcame inhibition caused by free histidine. In the case of Zn^++^, the presence of the cation alone increased both reactions by up to 50% above control, and virtually abolished the inhibition caused by histidine, with recoveries of 90 – 100% of the activity measured in the presence of Zn^++ ^alone. In the case of Co^++^, the cation alone increased the PDE activity by 50% and the LPLD activity by 300%; however, histidine still inhibited PDE by ~70% and LPLD by 50% compared to adding Co^++ ^alone. Zn^++^, therefore, appears to be the most effective cation in reversing the inhibition by histidine. It should also be noted that none of the cations at the levels tested had any inhibitory effects upon the enzymatic reactions of ATX. Histidine therefore inhibits a process that is required for the hydrolysis of both nucleotides and phospholipids. This may be a rate-limiting cation-dependent step in their common reaction mechanism [7].

Since Zn^++ ^reversed the inhibitory effect of histidine upon ATX enzymatic reactions, we tested its effect upon the histidine-induced inhibition of ATX- and LPA-stimulated motility. As shown in Fig. 3C for A2058 cells, 20 mM histidine, 0.25 mM Zn^++^, or both together had no effect upon LPA-induced motility. In contrast, ATX-induced motility was affected in a more complex manner. Histidine (20 mM) alone inhibited ATX-induced motility by ~65%, while 0.25 mM Zn^++ ^alone increased this same motility by ~30%. When these same concentrations of histidine and zinc were added together to an ATX-stimulated motility assay, the histidine inhibition was largely reversed with greater motility than seen with ATX alone, though not quite up to levels seen with ATX + Zn^++^. However, statistical analysis of these results revealed no statistically significant difference between ATX + Zn^++ ^+ histidine vs. ATX + Zn^++^. In contrast, there was a statistical significance between ATX + Zn^++ ^+ histidine vs. ATX + histidine (p < 0.001).

**Figure 4:**
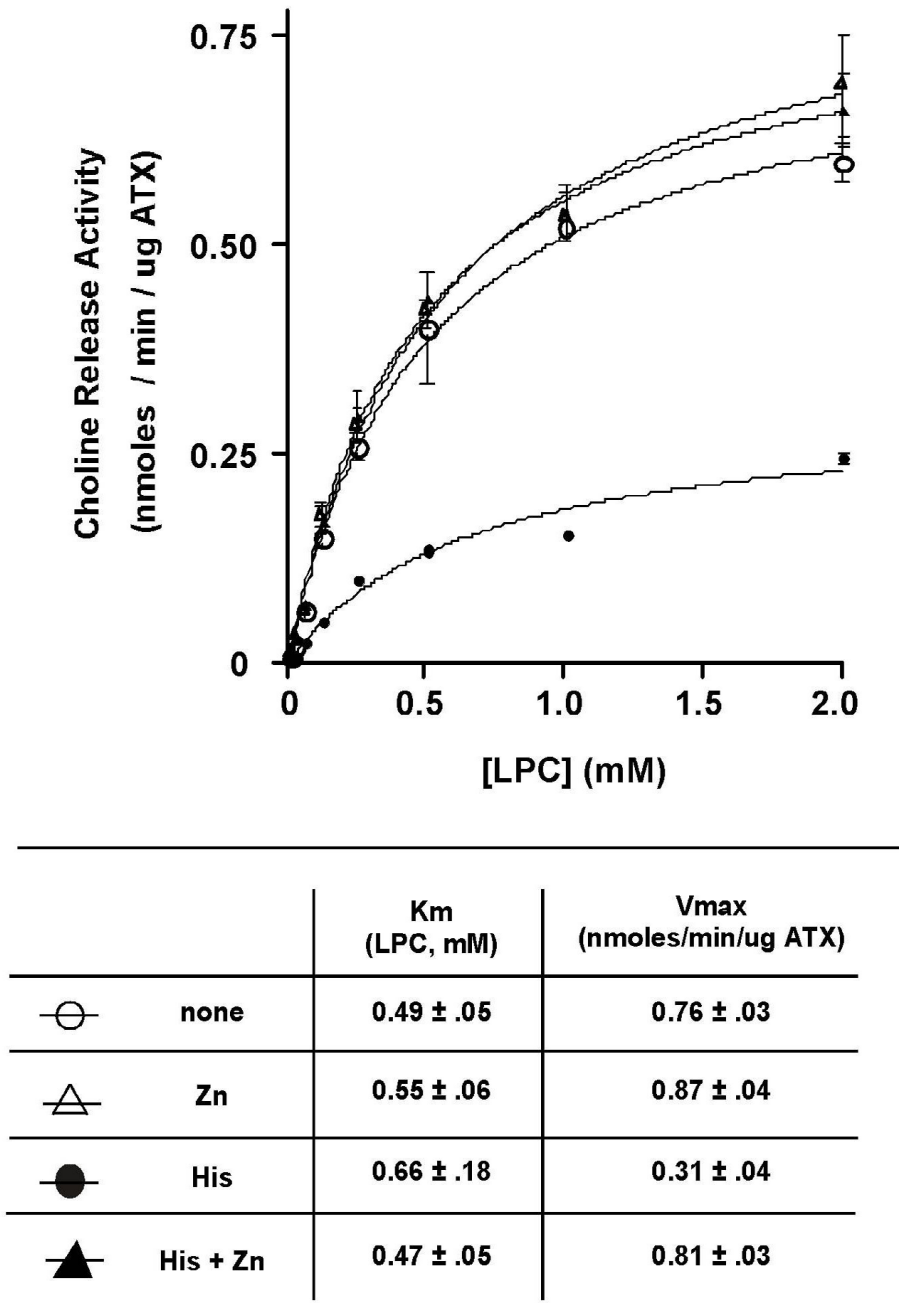
**Reaction rate vs. substrate concentration curves in the presence of L-histidine or zinc **LPLD determinations were carried out in the presence of 10 mM L-histidine or 0.25 mM Zn^++^, as indicated. Curves were analyzed with GraphPad Prism (San Diego, CA) to calculate Km and Vmax, as shown.

Fig. 4 shows a concentration curve of the ATX-catalyzed LPLD reaction with LPC as substrate, both with and without addition of 10 mM histidine. The two reactions had Km values (0.49 ± 0.05 mM untreated vs. 0.66 ± 0.18 mM with histidine) that were not significantly different from each other; however, Vmax was reduced more than 50% by the addition of histidine. Also shown are the effects of 0.25 mM Zn^++^, alone or in addition to histidine. Zn^++ ^alone had no significant effect on the Vmax or Km of the reaction. In the presence of histidine, Zn^++ ^reversed the inhibition, restoring Vmax to untreated levels. It might be noted that the pattern of inhibition seen when histidine is added is typical of a non-competitive inhibitor.

### Effect of Chelating Agents upon the ATX-stimulated Reaction

Because the histidine-induced inhibition of ATX activities could be reversed by addition of appropriate metal ions (i.e., Zn^++^), the possibility arose that the imidazole ring of histidine acts as a weak chelation agent, competing with the enzymatic active site for binding to the metal ion. EDTA has been shown to inhibit the nucleotide phosphodiesterase and pyrophosphatase activities of PC-1/NPP1 [19]. We, therefore, utilized EDTA, as well as the metal chelating agent 1,10-phenanthroline, in order to examine their effect upon the LPLD and PDE activities of ATX. We also tested the reversibility of their effects with Ca^++ ^and with Zn^++^.

**Figure 5:**
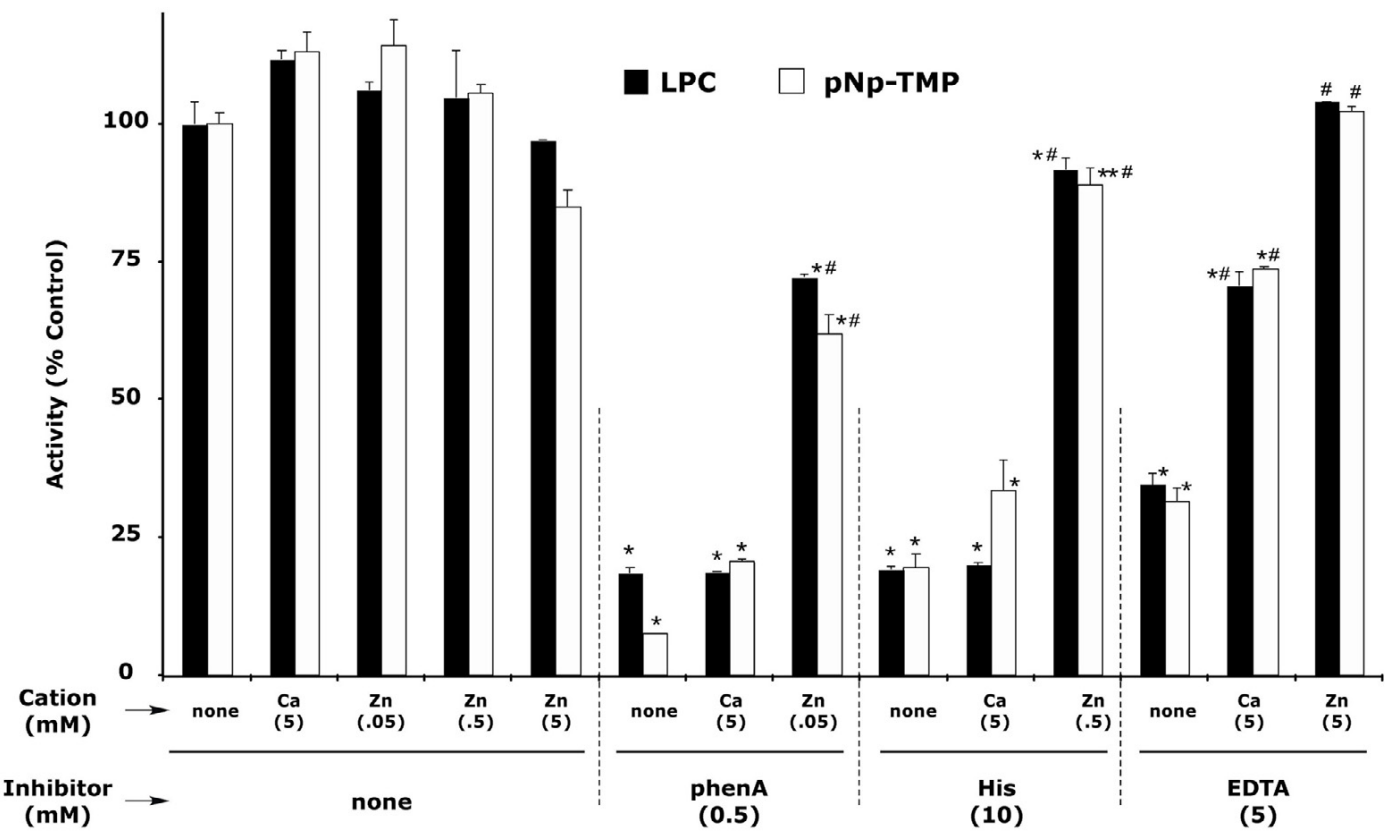
**Effect of chelating agents on ATX's LPLD activity **The LPLD activity of ATX was compared in the presence of 0.5 mM phenanthroline A (phenA), 5 mM EDTA, or 10 mM L-histidine. The effect of adding Zn^++ ^or Ca^++ ^was compared for each chelating agent. Results are expressed as Mean ± SEM. Statistical analysis of results was performed utilizing GraphPad Prism (San Diego, CA). Data that is significantly different from untreated control is shown by: * (p < 0.001), ** (p < 0.05); recovery with metal cations is shown as statistically different from appropriate inhibitor-treated samples: # (p < 0.001).

In a series of preliminary experiments (data not shown), we found that, under the conditions of our enzymatic assays, addition of 10 – 20 mM Na_4_-EDTA resulted in profound inhibition of both LPLD and PDE activities with an IC50 of approximately 5 mM. We also found that 5 mM Na_4_-EDTA required very nearly equimolar concentrations of metal cations to reverse its inhibitory effects. As shown in Fig. 5, addition of equimolar concentration of Zn^++ ^resulted in essentially complete recovery of activity (range 90 – 110% recovery) for both LPLD and PDE activities, while an equimolar concentration of Ca^++ ^resulted in ~75% recovery of control activity.

Addition of 0.5 mM 1,10-phenanthroline also profoundly inhibited the LPLD activity of ATX (Fig. 5). The IC50 for 1,10-phenanthroline in this reaction was approximately 0.25 mM (data not shown). As has been reported previously, a significant recovery from 1,10-phenanthroline-induced inhibition requires less than equimolar concentrations of Zn^++ ^[20]. Addition of 0.05 mM Zn^++ ^resulted in a 70 – 80% recovery compared to control values, while adding up to 5 mM Ca^++ ^had no significant effect upon phenanthroline-induced inhibition of activity. As can be seen in Fig. 5, the pattern of recovery from 1,10-phenanthroline-induced inhibition is similar to the pattern seen with histidine-induced inhibition of LPLD.

Testing the effect of these chelating agents on motility was problematic because chelation agents have multiple, complex effects upon living cells. For example, Na_4_-EDTA treatment killed our responder cells (A2058) in about 15 – 20 min, and 1,10-phenanthroline inhibited cellular adhesion at concentrations utilized for inhibition of LPLD and PDE activity. Therefore, we were unable to obtain motility data in the presence of these agents.

### Effects of Histidine Analogs upon the LPLD Reactivity of ATX

**Figure 6:**
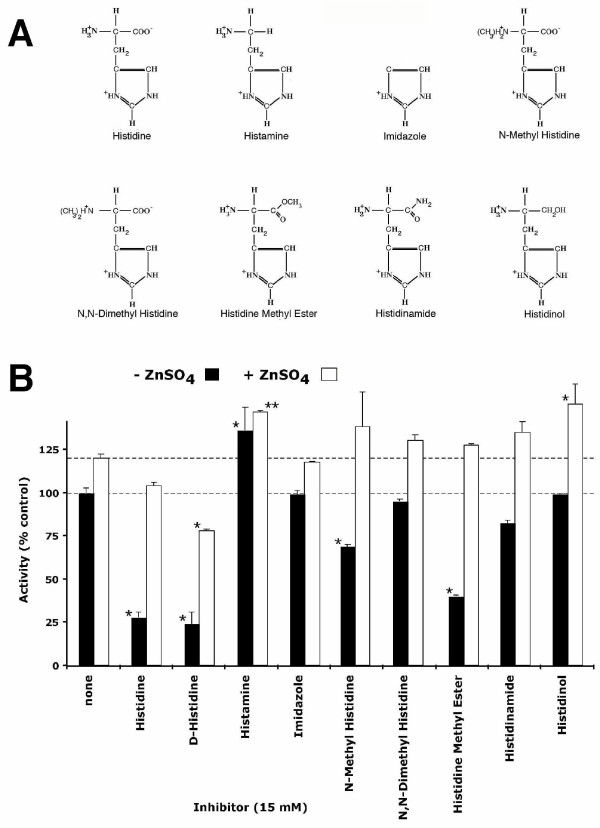
**Effect of histidine analogs on LPLD activity **A.) Chemical structure of histidine analogs compared. B.) Histidine analogs (15 mM) were added to the LPLD assay and analyzed for their effect on LPLD activity with (white bars) or without (black bars) addition of 1.0 mM Zn^++^. Results are shown as Mean ± SEM. Means, for each histidine analog vs. appropriate untreated control, were analyzed utilizing ANOVA/Tukey's post-test (GraphPad Prism, San Diego, CA): * (p < 0.001), ** (p < 0.01).

A number of histidine-derived agents, as well as histamine and imidazole (Fig. 6A), were tested for their effects upon the LPLD activity of ATX. Since 15 – 20 mM L-histidine gave similar levels of inhibition, all reactions were carried out in the presence of 15 mM inhibitor.

As seen in Fig. 6B, D-histidine and L-histidine are the most effective inhibitors of the ATX LPLD activity, resulting in approximately 75% reduction. They were not statistically significant from each other. Nearly as inhibitory was histidine methyl ester, which has an esterified alpha carboxyl group and which reduced LPLD activity by 65%. Other compounds with substitutions on the alpha carboxyl group, histidinamide and histidinol, were markedly less effective as inhibitors. Histidinol, in which the carboxyl group is reduced to a hydroxy-methyl group, was not statistically different from untreated control reactions. Histidinamide, with amidylation of the alpha carboxyl group, was mildly inhibitory, reducing activity ~20%. In contrast, histamine, which lacks the alpha carboxyl group, altogether, was slightly stimulatory to LPLD activity. Histidine analogs with methyl groups added to the alpha nitrogen were also less active than histidine itself. N, N-dimethyl histidine was not statistically different from untreated control, while N-methyl histidine, resulted in just a 30% reduction in LPLD activity. Surprisingly, at the tested concentrations, the metal-binding agent imidazole had no effect on the LPLD activity of autotaxin. Likewise, amino acids that resemble histidine without its imidazole ring (e.g., glycine and L-alanine) also lack inhibitory activity (data not shown).

Among the five histidine analogs found to inhibit ATX LPLD activity, the relative inhibitory activity is: D-histidine = L-histidine > histidine methyl ester >> N-methyl histidine > histidinamide. The effects of all of these agents are reversed by zinc. In contrast, glycine, L-alanine, imidazole, histidinol, and N, N-dimethyl histidine had no significant effect on activity; and histamine appeared to be slightly stimulatory. Most of these histidine analogs, including D-histidine, histidine methyl ester, histidinamide, N, N-dimethyl histidine, and histamine, were toxic to cells. N-methyl histidine resulted in reduced adhesion to our gelatin-coated membranes, precluding an effective motility assay. Only imidazole could easily be tested for its effect on ATX-stimulated motility. At 10–20 mM concentrations, imidazole had no significant effect on ATX-stimulated motility (data not shown).

## Discussion

We have shown that L-histidine, at concentrations that are innocuous to cells, can inhibit the motility-stimulating action of ATX upon two tumor cell lines derived from human melanoma (A2058) and human ovarian carcinoma (SKOV-3). This inhibition is largely abrogated by addition of 0.25 mM Zn^++ ^to the motility assays. Pre-incubation of ATX and LPC in the presence of L-histidine, followed by heat-killing the ATX, also resulted in much decreased activity, suggesting that L-histidine inhibits ATX-stimulated motility by ablating its LPLD activity. This hypothesis was in fact confirmed both by direct enzymatic studies in which L-histidine inhibited both PDE and LPLD activities and by the failure of ATX, in the presence of L-histidine, to produce an inhibitor of LPA-stimulated motility. Again, addition of Zn^++ ^abrogated this inhibition of enzyme activity. These data provide further evidence that the LPLD activity of ATX, which results in the production of the mediator LPA, is essential for ATX stimulation of cellular motility.

The role of metal cations in the activation of ATX was further investigated by first comparing the capacity of Ca^++^, Mg^++^, Zn^++ ^and Co^++ ^to reverse the histidine-induced inhibition of ATX enzymatic activities. Neither Ca^++ ^nor Mg^++ ^reversed the inhibitory effect of histidine, whereas Co^++ ^alone stimulated a 50% increase in PDE and a 300% increase in LPLD activities of ATX. Histidine partially reversed both of these Co^++^-stimulated increases: PDE activity was reduced to below uninhibited levels of product while LPLD activity, though reduced by 50% compared to treatment with Co^++ ^alone, remained at levels above untreated controls. The mechanism by which cobalt activates ATX remains unexplained; however, Co^++ ^has long been reported to replace one or more zinc cations in a variety of zinc-bound metalloenzymes [21-23], sometimes with a notable increase in activity [24,25].

In contrast, Zn^++ ^at concentrations as low as 0.25 mM abrogated the inhibitory effects of 10 mM histidine upon both the PDE and LPLD activities of ATX. This 40:1 ratio of ligand to cation appears to be inconsistent with a simple stoichiometric coordination of the cation with the metal-binding imidazole moiety. This would predict that the maximum number of ligand groups to coordinate with a Zn^++ ^would be six. Our result is, therefore, difficult to explain as simple chelation in solution between cations and histidine. Perhaps, Zn^++ ^acts upon ATX in a manner that blocks the entry of histidine into the active site. For example, Zn^++ ^might coordinate with an electronically available locus in proximity with the active site of ATX or it might induce conformational changes in ATX that lowers the affinity of free histidine for the active site. Another possibility is that Zn^++ ^replaces another metal ion, resulting in a binding conformation with a lower affinity for L-histidine. Interestingly, 0.25 mM Zn^++ ^appeared to have a slightly stimulatory effect upon ATX-induced motility, but not LPA-induced motility. This suggests that Zn^++ ^might act in a way that stabilizes ATX in an active configuration.

Many members of the alkaline phosphatase superfamily of metalloenzymes have Zn^++ ^or Zn^++ ^plus Mg^++ ^incorporated into their catalytic site, although a number of other metal requirements have been documented [14,26]. The chelation agents, Na_4_-EDTA and 1,10-phenanthroline both inactivate ATX, confirming that it is a metalloenzyme. Na_4_-EDTA complexes with a variety of cations, including transition metals as well as alkali and alkaline earth metals, and is thought to inactivate metalloenzymes by removing the metal cation from its binding site. Under the conditions of our assays, the IC50 of EDTA was ~5 mM. This inhibition was completely reversed by addition of equimolar Zn^++ ^and significantly reversed (about 75% recovery) by addition of equimolar Ca^++^. In contrast, 1, 10-phenanthroline has more complicated and diverse mechanisms of action. When utilized with metalloenzymes, it can form mixed complexes with the metal ions as well as other cationic sites on the protein, resulting in inactivation of the enzyme but not necessarily removal of the metal cation [27]. Like histidine, 1,10-phenanthroline requires less than equimolar concentrations of Zn^++ ^(approximately 1:10) to reverse its inhibitory effect on ATX. Based on all of our data with metal cations, it appears that Zn^++^, Co^++ ^and perhaps Ca^++ ^can function as the metal moiety in at least one of the two metal binding sites [15] of the active metalloenzyme ATX, although we do not know the predominant metal cations in the native enzyme.

In order to determine what portion of the histidine molecule was responsible for its inhibitory activity, we utilized a number of commercially available histidine analogs. These analogs were predominantly substituted on the alpha carboxyl and alpha amino groups of histidine; but they also included histamine, which lacks the alpha carboxyl group altogether, and imidazole, the weakly chelating ring that makes up the major portion of the histidine side chain. Except for D-histidine, none of the other imidazole-containing compounds were as inhibitory as L-histidine. Among the 5 agents found to have significant inhibitory activity, the relative potency is as follows: D-histidine = L-histidine > histidine methyl ester >> N-methyl histidine > histidinamide. Methylation of the alpha amino group of histidine resulted in a stepwise loss of potency, with a single methylation (N-methyl histidine) resulting in about a 40% reduction in activity and a double methylation (N, N-dimethyl histidine) not significantly different from untreated control reactions. Since these two compounds should retain the positive charge properties of the native alpha amino group, this could be a steric effect. Similarly, change in the carboxyl group (particularly amidylation or reduction) also decreased the inhibitory potency, though that of esterified histidine methyl ester was only slightly reduced. Interestingly, imidazole itself, a moiety postulated to coordinate with the cation at the active site of ATX, was not effective at the tested concentrations. Likewise, amino acids, which lacked imidazole on their side chains (e.g. glycine and L-alanine), were also ineffective as inhibitors. Clearly, three functional groups of histidine are required for full inhibitory activity: the alpha amino group, the alpha carboxyl group, and the metal-binding imidazole side chain. Recent work with Zn^++^-binding groups in matrix metalloproteinase inhibitors indicated that slight changes to the Zn^++^-binding groups can result in major changes in efficacy of inhibition [28], suggesting an approach for developing more potent, and perhaps highly specific, pharmacologic inhibitors.

ATX, the major source of plasma LPA, has been implicated in a number of physiological and disease-related processes [29], including tumor metastasis [30] and angiogenesis [31]. A pharmacologic agent that inhibits ATX activity could have major therapeutic implications. Other than the highly non-specific chelation agents [18], L-histidine is the first reported inhibitor that acts on the LPLD activity of ATX rather than acting on its downstream activation cascade. This inhibition, requiring millimolar concentrations of L-histidine, is reversible by Zn^++ ^metal cations. Our data suggest that the L-histidine-induced inhibition of the ATX enzymatic activities is predominantly non-competitive, i.e. Vmax is decreased significantly, while Km is not significantly affected. Furthermore, the mechanism of action of L-histidine does not appear to be simple stoichiometric chelation of the metal cations within a metalloenzyme but is more likely a complex interaction with a variety of moieties, including the metal cation, at or near the active site. At concentrations required for an inhibitory effect, most available histidine analogs resulted in loss of cell viability. Free L-histidine, therefore, appears to be unique in its ability to inhibit LPLD, and hence regulate a major source of LPA, in living systems. Since LPA has significant pathological effects, the regulation of its formation is of considerable interest. Potential problems with L-histidine as a therapeutic agent include its lack of potency and its conversion *in vivo *to histamine. While there are known inhibitors of histidine decarboxylase, such as the polyphenols of green tea [32], potent, stable, and non-toxic analogs of histidine would appear to be better therapeutic agents.

## Conclusion

L-histidine inhibits the LPLD activity of ATX at millimolar concentrations, reducing its capacity to produce its major mediator, LPA.

## Methods

### Reagents

LPA (18:1) and S1P were purchased from Biomol Research Laboratories, Inc. (Plymouth Meeting, PA). LPC (18:1), SPC, N-ethyl-N-(2-hydroxy-3-sulfopropyl)-m-toluidine (TOOS), 4-aminoantipyrene (4-AAP), horseradish peroxidase, choline oxidase, p-nitrophenyl-TMP, L-histidine, D-histidine, imidazole, histamine, histidinamide, histidine methyl ester, N,N-dimethyl histidine, histidinol, Na_4_-EDTA, and 1,10-phenanthroline were from Sigma-Aldrich (St Louis, MO). N-methyl histidine was from Bachem Bioscience (King of Prussia, PA).

### Cell Lines

A2058 [33] and SKOV-3 cells were maintained in Dulbecco's Modified Essential Medium (DMEM) supplemented by 2 mM glutamine, 1X penicillin/streptomycin and 10% (v/v) heat-inactivated fetal bovine serum. SKOV-3 cells (ATCC # HTB-77) were purchased from American Type Culture Collection (Manassas, VA).

### ATX Preparations

Highly purified recombinant human ATX (vATX), cloned from an MDA-MB-435 cell library, was prepared from a Vaccinia viral lysate, as described previously, through the concanavalin A-agarose step [34].

### *In vitro *Motility Assays

Cells were detached from their flasks with a brief exposure to 0.05% trypsin and 0.02% versene and then resuspended at 2 × 10^6 ^cells/ml in DMEM supplemented with 1 mg/ml bovine serum album (DBA). Ten min before the start of the assay, appropriate concentrations of potential inhibitors were added to the different treatment groups. Chemotaxis was assayed as described previously in detail [35], utilizing gelatin-coated membranes for A2058 cells and Type IV collagen-coated membranes for SKOV-3 cells. Chemotaxis chambers were incubated 3 hr for A2058 cells and 5 hr for SKOV-3 cells. Migrated cells were fixed and stained, then quantified by cell counting under light microscopy at 200X (SKOV-3 cells) or 400X (A2058 cells).

### Enzymatic Activity Assays

Enzyme activities were determined using a modification of the previously described assays [9]. Briefly, a 5 ml aliquot of vATX (adjusted to give ≈ 6 pmoles vATX/reaction) was incubated (50 μl reaction volume) with either 1 mM pNp-TMP or, alternatively, with 1 mM LPC, for 45 min at 37°C in DBA. DBA was utilized in order to mimic the conditions of motility assays. For assays with histidine and its analogues, stock solutions were prepared and adjusted to physiological pH with NaHCO_3 _or HCl, as appropriate. The inhibitor was added to the reaction mixture; then, reactions were initiated by adding ATX.

For determination of 5'-nucleotide PDE activity, reactions were stopped by the addition of 450 μl 0.1 N NaOH and the nitrophenol product was detected by reading the absorbance at 410 nm (A410 × 64 = nmoles). In order to determine LPLD activity, released choline was detected as follows: a 450 μl cocktail containing 50 mM Tris-HCl (pH 8), 5 mM CaCl_2_, 0.3 mM N-ethyl-N-(2-hydroxy-3-sulfopropyl)-m-toluidine (TOOS), 0.5 mM 4-aminoantipyrene (4-AAP), 5.3 U/ml horseradish peroxidase, and 2 U/ml choline oxidase was added to the 50 μl reaction mixture and incubated for 20 min at 37°C. Absorbance was read at 555 nm and converted to nmoles of choline by comparison to a choline standard curve (A555 × 17 = nmoles).

## List of Abbreviations Used

**ATX **Autotaxin

**DBA **DMEM with 1% bovine serum albumin

**DMEM **Dulbecco's Modified Essential Medium

**LPA **Lysophosphatidic acid

**LPC **Lysophosphatidylcholine

**LPLD **Lysophospholipase D

**NPP **Nucleotide pyrophosphatase and phosphodiesterase

**PDE **Phosphodiesterase

**pNp-TMP **p-Nitrophenyl-TMP

**S1P **Sphingosine-1-phosphate

**SPC **Sphingosylphosphorylcholine

## Authors' Contributions

TC carried out the enzymatic studies, EK performed cellular studies with A2058 cells, MP performed cellular studies with SKOV-3 cells, and RWB carried out background studies and fine-tuned methodology. ES originated the idea of studying the effect of L-histidine on LPLD activities. LAL and MLS analyzed and interpreted data. MLS drafted and coordinated the manuscript between authors. All authors participated in reading and refining the manuscript drafts.
